# Supplementary material for: Association of handgrip strength asymmetry and weakness with successful aging among older adults in China
Source: PLoS One. 2026 Jan 14;21(1):e0329248. doi: 10.1371/journal.pone.0329248 (PMC12803466; doi:10.1371/journal.pone.0329248)

**Supplementary Table S1.**

|  | Model 1 | | Model 2 | |
| --- | --- | --- | --- | --- |
|  | OR (95% CI) | *P*-value | OR (95% CI) | *P*-value |
| **Male** |  |  |  |  |
| HGS asymmetry (Reference = No) |  |  |  |  |
| Yes | 0.552 (0.399,0.765) | <0.001 | 0.559 (0.403,0.776) | 0.001 |
| HGS weakness (Reference = No) |  |  |  |  |
| Yes | 0.497(0.330,0.749) | 0.001 | 0.636(0.416,0.974) | 0.037 |
| HGS group (Reference = Normal) |  |  |  |  |
| Asymmetry only | 0.564(0.391,0.814) | 0.002 | 0.579(0.400,0.838) | 0.004 |
| Weakness only | 0.491(0.280,0.862) | 0.013 | 0.643(0.359,1.150) | 0.136 |
| Both | 0.329(0.185,0.585) | <0.001 | 0.418(0.233,0.752) | 0.004 |
| HGS asymmetry severity (Reference = Normal) |  |  |  |  |
| Mild asymmetry | 0.592(0.401,0.874) | 0.008 | 0.599(0.404,0.887) | 0.010 |
| Moderate asymmetry | 0.654(0.374,1.143) | 0.136 | 0.695(0.395,1.221) | 0.206 |
| Severe asymmetry | 0.361(0.181,0.723) | 0.004 | 0.396(0.197,0.797) | 0.009 |
| **Female** |  |  |  |  |
| HGS asymmetry (Reference = No) |  |  |  |  |
| Yes | 1.562(1.119,2.182) | 0.009 | 1.525(1.089,2.136) | 0.014 |
| HGS weakness (Reference = No) |  |  |  |  |
| Yes | - | - | - | - |
| HGS group (Reference = Normal) |  |  |  |  |
| Asymmetry only | 0.640(0.458,0.894) | 0.009 | 0.656(0.468,0.919) | 0.014 |
| Weakness only | - | - | - | - |
| Both | - | - | - | - |
| HGS asymmetry severity (Reference = Normal) |  |  |  |  |
| Mild asymmetry | 0.881(0.603,1.287) | 0.514 | 0.873(0.596,1.279) | 0.485 |
| Moderate asymmetry | 0.590(0.324,1.073) | 0.084 | 0.620(0.340,1.133) | 0.121 |
| Severe asymmetry | 0.289(0.144,0.580) | <0.001 | 0.307(0.153,0.619) | 0.001 |

HGSweakness is not present in the female group

**Supplementary Fig.S1**


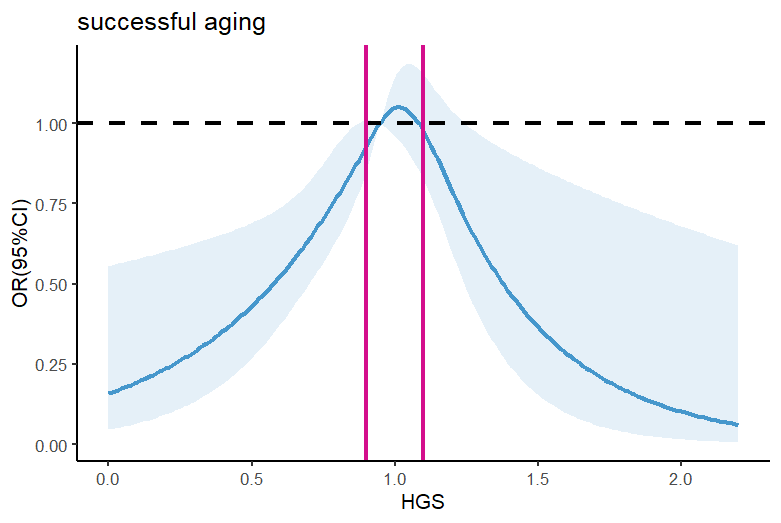

Supplement: S1 Table — (DOCX) [file pone.0329248.s001.docx]
