# Supplementary material for: Association of handgrip strength asymmetry and weakness with successful aging among older adults in China
Source: PLoS One. 2026 Jan 14;21(1):e0329248. doi: 10.1371/journal.pone.0329248 (PMC12803466; doi:10.1371/journal.pone.0329248)
Supplement: Attachment — Submitted filename: Notes_reviewer.docx [file pone.0329248.s002.docx]

**Association of handgrip strength asymmetry and weakness with successful aging among older adults in China**

**Abstract**

**Overall Assessment**

The manuscript *“Association of handgrip strength asymmetry and weakness with successful aging among older adults in China”* investigates the relationship between handgrip strength (HGS) asymmetry/weakness and successful aging (SA) using nationally representative data from CHARLS. The topic is highly relevant, and the study has the potential to contribute meaningfully to the literature on aging and physical function. The dataset is large and robust, and the statistical approach is multifaceted, including logistic regression, subgroup analysis, and restricted cubic spline (RCS) modeling.

Nevertheless, the manuscript requires major revisions before it can be considered for publication. In its current form, it suffers from issues of methodological transparency, definitional clarity, language precision, and depth of discussion.

**Abstract**

- The statement *“The role of handgrip strength (HGS) asymmetry and weakness in successful aging remains unclear”* is too strong. The introduction and discussion themselves cite prior studies showing associations between HGS and SA or related outcomes. I recommend softening this claim (e.g., “remains insufficiently understood” or “requires further clarification”).

**Definition and Operationalization of SA**

- The criteria for SA are drawn from Rowe and Kahn, but the rationale for their operationalization is insufficiently explained.
- The prevalence of SA in this study (6.3%) is much lower than previously reported rates (~22%). This discrepancy should be carefully explained (e.g., cultural, methodological, or sample-related factors).
- For the “active social participation” component, it is unclear whether frequency, intensity, or duration were assessed, or if the indicator was binary (any participation in the past month). Greater clarity is needed.

**Keywords**

- All keywords are essentially identical to the manuscript title, except “CHARLS.” More diverse and meaningful keywords should be added (e.g., frailty, cognitive function, China, aging population).

**Measurement of HGS**

- The method of identifying the dominant hand is not specified (self-report, test, observation?). This should be clarified.
- The study uses absolute HGS cut-offs (<28 kg for men, <18 kg for women). However, relative HGS (adjusted for body weight) is commonly used in gerontology and may provide more accurate information. The authors should justify their choice of absolute values and cite recent consensus (e.g., EWGSOP2). Two people with the same absolute strength but different weights may have different risk profiles.

**Statistical Analyses**

- Results of assumption checks for the applied statistical methods are missing. For example, logistic regression assumes linearity of the logit, and this is not addressed.
- Only two models are presented: Model 1 (crude) and Model 2 (fully adjusted). While this approach is not incorrect, it would be more informative to present stepwise adjustments. The rationale for the chosen modeling strategy should be explained.
- Sensitivity analyses are mentioned but not described in sufficient detail. A clear explanation of their design and execution is necessary.
- The RCS model shows an “N-shaped” relationship, but the interpretation is unclear. The term “N-shaped” is unconventional; a clearer description (e.g., inverted U-shaped or nonlinear association) is needed, along with biological or clinical interpretation.
- Some associations are only marginally significant (e.g., “weakness only”), yet they are interpreted as conclusive. Greater caution is warranted.

**Discussion**

- The discussion tends to be repetitive and sometimes speculative, restating results rather than deeply contextualizing them.
- The cross-sectional design is acknowledged but not sufficiently emphasized as a limitation. Stronger statements are needed regarding the inability to infer causality.

**Structure, Style, and Consistency**

- The terms “frailty” and “weakness” appear to be used interchangeably at times. These should be clearly distinguished.
- Figures and tables are generally useful, but some legends lack detail (e.g., Figure 3 should clearly explain the meaning of the red lines).
- Numerous small formatting inconsistencies are present (e.g., missing space before parentheses: “included(Fig. 1)”). These should be corrected.

**Conclusion**

The manuscript addresses an important and original research question, supported by a large and representative dataset. However, substantial revisions are required to improve methodological transparency, strengthen interpretation, and ensure clarity of presentation. The most critical weakness is the absence of reported assumption checks for the statistical tests applied (e.g., Binary logistic regression, rank-sum test and Pearson's chi-square test), which undermines confidence in the validity of the results. I therefore recommend major revision.
